# Simulation of TTT Curves for Additively Manufactured Inconel 625

**DOI:** 10.1007/s11661-018-4959-7

**Published:** 2019

**Authors:** G. LINDWALL, C.E. CAMPBELL, E.A. LASS, F. ZHANG, M.R. STOUDT, A.J. ALLEN, L.E. LEVINE

**Affiliations:** Material Measurement Laboratory, National Institute of Standards and Technology, 100 Bureau Drive, Gaithersburg, MD 20899; KTH Royal Institute of Technology, Brinellvgen 23, Stockholm, 10044, Sweden.; Material Measurement Laboratory, National Institute of Standards and Technology.; Material Measurement Laboratory, National Institute of Standards and Technology.; Material Measurement Laboratory, National Institute of Standards and Technology.; Material Measurement Laboratory, National Institute of Standards and Technology.; Material Measurement Laboratory, National Institute of Standards and Technology.; Material Measurement Laboratory, National Institute of Standards and Technology.

## Abstract

The ability to use common computational thermodynamic and kinetic tools to study the microstructure evolution in Inconel 625 (IN625) manufactured using the additive manufacturing (AM) technique of laser powder-bed fusion is evaluated. Solidification simulations indicate that laser melting and re-melting during printing produce highly segregated interdendritic regions. Precipitation simulations for different degrees of segregation show that the larger the segregation, i.e., the richer the interdendritic regions are in Nb and Mo, the faster the *δ*-phase (Ni_3_Nb) precipitation. This is in accordance with the accelerated d precipitation observed experimentally during post-build heat treatments of AM IN625 compared to wrought IN625. The *δ*-phase may be undesirable since it can lead to detrimental effects on the mechanical properties. The results are presented in the form of a TTT diagram and agreement between the simulated diagram and the experimental TTT diagram demonstrate how these computational tools can be used to guide and optimize post-build treatments of AM materials.

## I. INTRODUCTION

INCONEL 625 (IN625) is a solid solution-strengthened Ni-based superalloy characterized by high strength and good corrosion and oxidation resistance at high temperatures. These properties, in addition to excellent weldability and brazeability, make the alloy particularly interesting to the aerospace field. The weldability is also an indication that IN625 can be readily manufactured additively using, for example, the laser powder-bed fusion (L-PBF) technique.

The potential for additive manufacturing (AM) technologies to transform our current view of material and component design has resulted in AM gaining a lot of attention across multiple industries. This is especially true in the aerospace field where major efforts are being made to accelerate the industrialization of AM for use in low-volume production of complex parts using high-performance materials. Hence, AM IN625 has been researched extensively (e.g., References 1–7). For example, recent experimental work at the National Institute of Standards and Technology (NIST), USA, focused on L-PBF produced IN625 and the effect of different post-build heat treatments on the microstructure evolution.^[1–5]^ The extreme processing conditions during AM using L-PBF, with cooling rates as high as 10^6^ K/s, are known to result in large residual stresses in the as-built parts; thus, AM system providers recommend stress-relieving treatments for IN625 before the components are removed from the build plate.^[8]^

The high solidification rates followed by several heating and cooling cycles during the L-PBF process result in a very fine but highly segregated as-built microstructure^[1–3]^ which is in contrast to the coarser solidification microstructure seen in conventionally produced material. Therefore, the post-processing heat treatments recommended for wrought IN625 need to be altered to achieve the desired microstructure for the AM grades.

**Fig. 1—:**
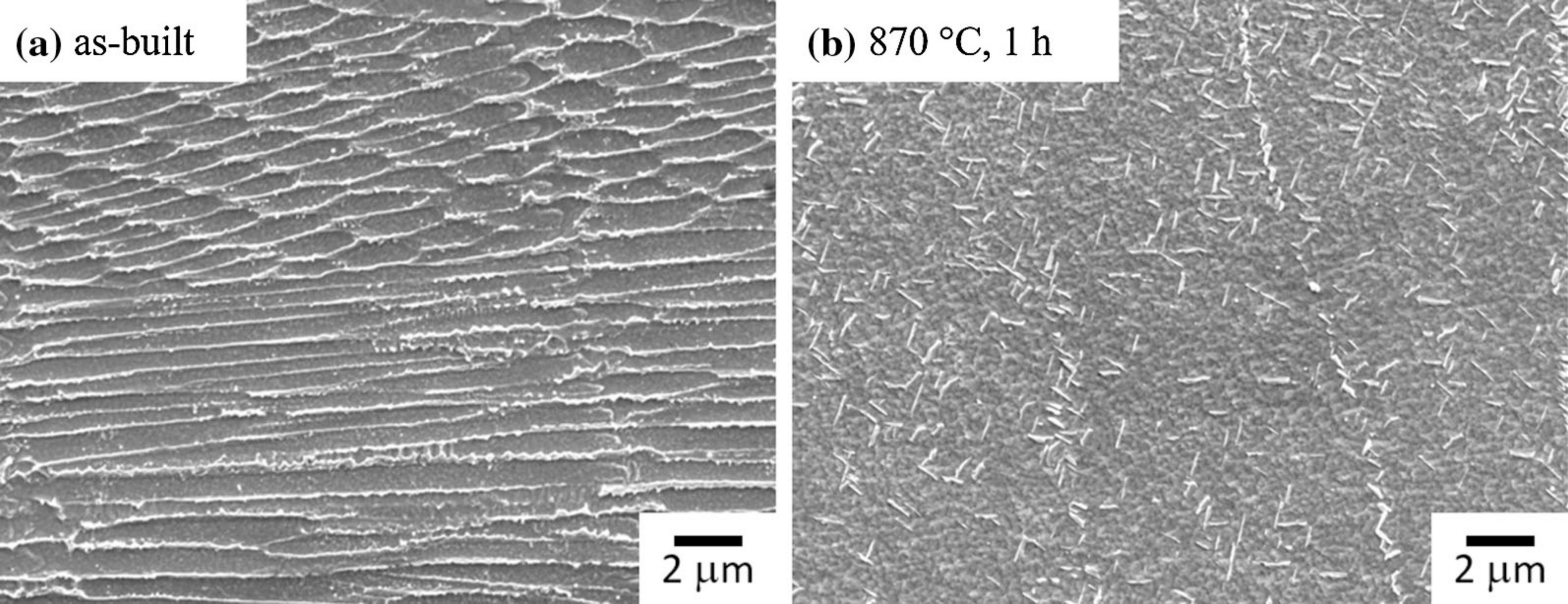
SEM image of L-PBF IN625 in (*a*) its as-built condition and (*b*) after heat treatment for one hour at 87 °C

**Fig. 2—:**
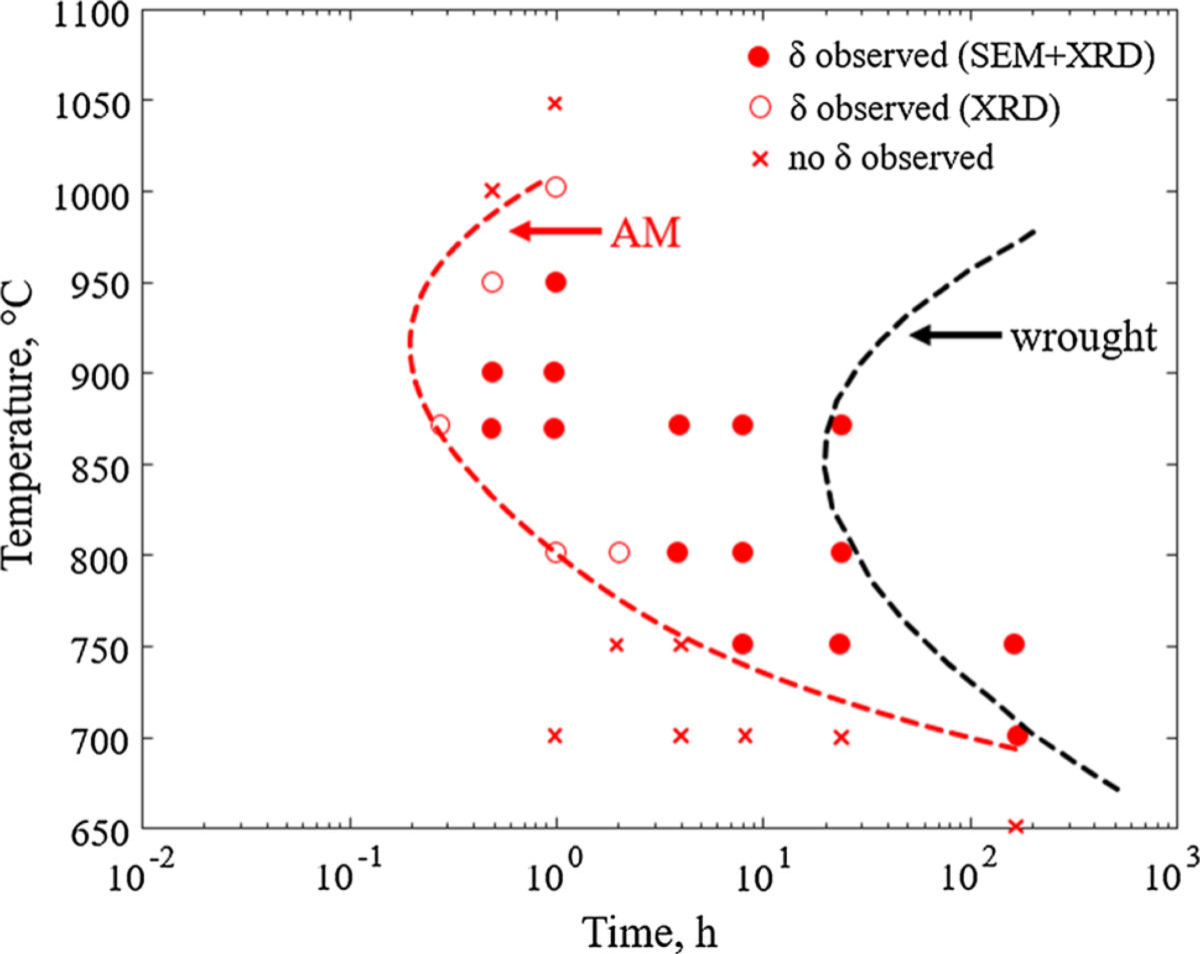
Experimentally determined TTT diagram for L-PBF IN625.^[3,5]^ The curves represent the time-temperature combinations that result in a volume fraction of ≈1 pct *δ*-phase in AM IN625 (red solid line) compared to wrought IN625 (black dashed line), from Ref. [9] (Color figure online).

Previously, Lass and Stoudt^[3,5]^ demonstrated that the precipitation kinetics during post-processing occurs at a shorter timescale in AM IN625 compared to wrought IN625. Their work demonstrated that the industry-recommended heat treatment promotes the formation of a significant fraction of the orthorhombic D0_a_ Ni_3_Nb *δ*-phase. They studied IN625 samples manufactured by L-PBF using scanning and transition electron microscopy (SEM/TEM) and X-ray diffraction (XRD) and concluded that approximately 2 vol pct *δ*-phase was formed after an hour at 870 °C (Figure 1). This should be compared to wrought IN625 where *δ*-phase is first detected after about 10 hours to 20 hours^[9,10]^ of heat treatment at 870 °C. The results for AM IN625 by Lass and Stoudt,^[3,5]^ are presented in terms of a time-temperature-transformation (TTT) diagram in Figure 2. The TTT curve (red dashed line) represents the time-temperature combinations for which roughly 1 vol pct *δ*-phase has formed. The filled circles in the figure indicate the time-temperature combinations for which *δ* was observed in SEM and XRD. The open circles indicate the time-temperature combinations for which *δ* was observed only with XRD, and thus correspond to lower volume fraction of *δ*-phase. In Figure 2, the measured TTT curve for *δ* formation in a wrought IN625 material^[9]^ is included for comparison (dashed black line). Accelerated *δ* precipitation has been observed during post-heat treatments of IN625 welds^[9,11,12]^ and rapidly solidified IN625 ribbons.^[13]^ Many of the physical processes in AM are very similar to those of welding, and similarities in the microstructural response are expected.

The observation of early formation of *δ*-phase in AM IN625 is also supported by *in situ* synchrotron scattering and diffraction experiments using combined ultra-small-angle X-ray scattering (USAXS), small-angle X-ray scattering (SAXS), and X-ray diffraction (XRD).^[4,5]^ There, the presence of distinct *δ*-phase peaks was observed within a 5-minute isothermal exposure at 870 °C. Since the *δ*-phase is known to have a detrimental influence on mechanical properties^[14–16]^ in conventional, wrought IN625, and is generally considered to be deleterious to materials’ in-service performance, its formation during post-treatments should be minimized.

The accelerated precipitation kinetics of the *δ*-phase in L-PBF IN625 is attributed to the segregated microstructure.^[3–5,17]^ Some studies also indicate that stresses may affect the precipitation kinetics in IN625^[10,18]^ although this effect most likely plays a lesser role compared to the segregation in AM IN625.^[3]^ In the current work, the working hypothesis is that the enhanced precipitation kinetics during stress-relieving of AM IN625 is mainly due to the compositional enrichments in certain regions caused by the segregation during solidification and reheating and cooling during the build process. Computational thermodynamics and kinetics are applied to evaluate the ability to use common computational tools to AM materials and to the optimization of post-build heat treatments. This comprises solidification simulations using the DICTRA module and precipitation simulations using the TC-PRISMA module, both within the Thermo-Calc software package*.^[19]^ The approaches use materials input data calculated using CALPHAD (CALculation of Phase Diagrams) databases, which make it possible to account for the multicomponent thermodynamic and kinetic aspects of the microstructure evolution. The results are compared and discussed based on conclusions drawn from experimental studies and presented in a time-temperature-transformation (TTT) diagram. In addition to published experimental data, volume fraction data obtained by *in situ* synchrotron diffraction experiments are compared to.

## II MATERIAL

The composition of the as-received IN625 powder used in the experimental investigation is listed in Table I. IN625 was designed for the use at high temperature where it will be a solid solution-strengthened alloy with no significant microstructure features other than the *γ* (fcc: face-centered-cubic) matrix. At lower temperatures, however, different phases may precipitate depending on the process conditions.

Conventionally produced IN625 is usually solution treated at temperatures between 1093 °C and 1204 °C^[20]^ and complete stress-relief is achieved when heated to 870 °C.^[20]^ This temperature (870 °C) is also currently the industry-recommended stress-relieving temperature for an as-built IN625 part on the build plate when manufactured using L-PBF, *i.e.,* 1 hour at 870 °C.

**Fig. 3—:**
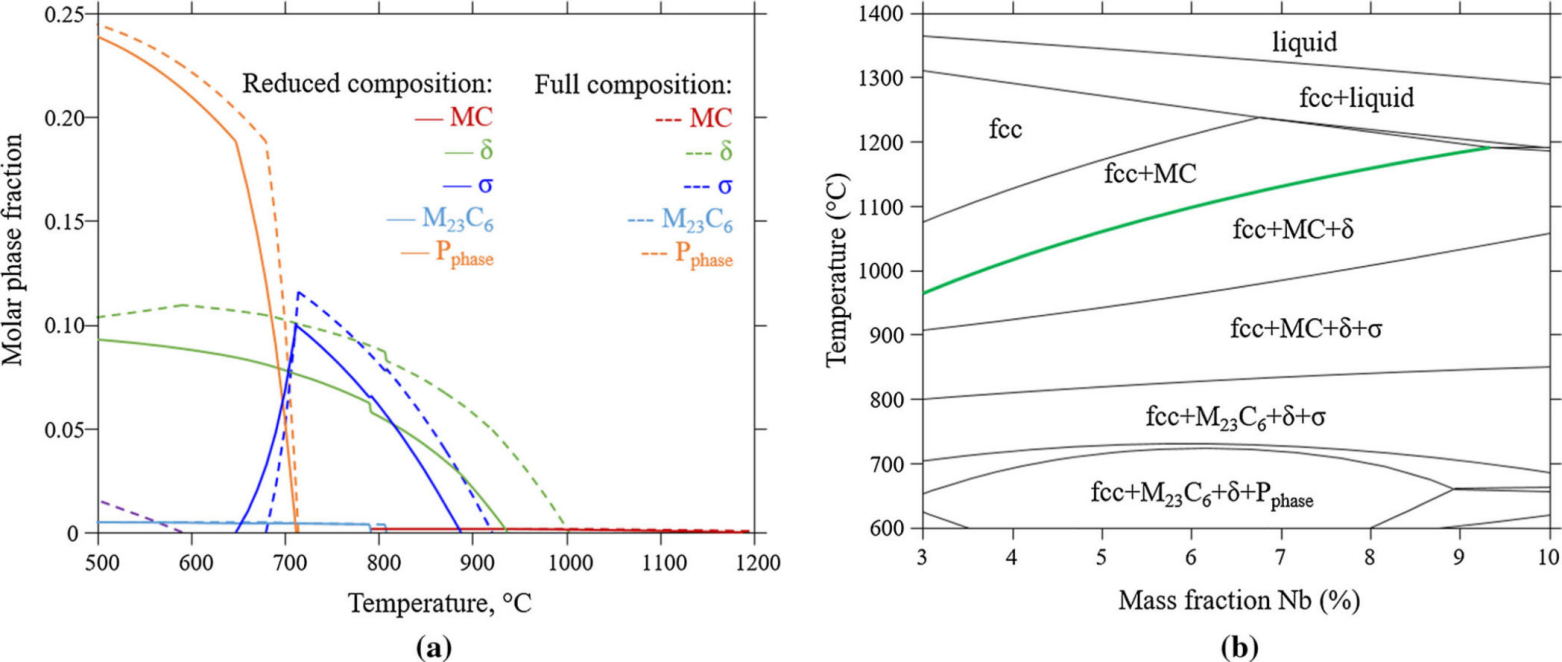
(*a*) Calculated equilibrium phase fraction as a function of temperature for the reduced composition (solid lines, composition: 0.02 pct C,20.7 pct Cr, 0.72 pct Fe, 3.75 pct Nb, 8.83 pct Mo) used for the DICTRA simulations and the full composition (dashed lines), see Table I. (*b*) Calculated isopleth for varying Nb compositions. The green line shows how the d solvus increases with increasing Nb. The thermodynamic information is taken from the commercial thermodynamic database TCNI8.[18]

We used the commercial thermodynamic database TCNI8/Ni superalloys^[21]^ to calculate equilibrium phase fractions. For the computational work presented in the following sections, a reduced (simplified) composition is used in order to reduce the simulation time. The simplified composition includes C and the elements with a mass fraction of>0.5 pct, *i.e*., Cr, Fe, Mo, Nb and Ni. The reduced composition is 0.02 pct C, 20.7 pct Cr, 0.72 pct Fe, 3.75 pct Nb, 8.83 pct Mo. Here, we note that a lower C content is given in the powder specification but was increased to 0.02 pct to avoid numerical problems in the precipitation simulations described in the following sections. To validate this composition, we compared the equilibrium phase fractions calculated using this reduced composition with the nominal composition shown in Table I. The result is shown in Figure 3(a). We found that at a typical solutionizing temperature (≈1100 °C to 1200 °C), a small fraction of MC carbide is in equilibrium with the *γ*-matrix. At lower temperatures, a number of different phases become stable; e.g., at the stress-relieving temperature, 870 °C, MC, *δ*, and *σ* are predicted to be in equilibrium with the *γ*-matrix.

The calculation in Figure 3(a) predicts somewhat lower phase fractions of *σ* and *δ* compared to the calculation for the actual composition. In particular, the temperature range for the *δ* stability appears sensitive to composition variations; *e.g*., the solvus temperature is lowered by approximately 60 °C when Al, Mn, Si, and Ti are excluded. This should be kept in mind when comparing calculation results for the reduced composition with experimental observations in the following part of this paper.

Thermodynamic equilibrium represents the final state of a system at given conditions and is often not fully reached during typical processing times. Nevertheless, it provides information about what the system is aiming towards. In the case of the IN625 composition, an equilibrium fraction of *δ* is expected for a wide temperature range. The interest lies in how long it would take to reach the final state, what other phases that may precipitate meanwhile and how this is altered due to compositional or process condition variations. From a thermodynamic perspective, sensitivity to compositional variations is exemplified by the calculated isopleth for varying Nb compositions (Nb replacing Ni) shown in Figure 3(b). The *δ* solvus temperature extends significantly to higher temperature when the Nb composition increases.

## III COMPUTATIONAL TOOLS

### A Solidification Simulations

The microsegregation during solidification of IN625 is simulated using DICTRA.^[19]^ DICTRA allows for simulation of diffusion-controlled phase transformations in one dimension (1D) assuming local equilibrium at a sharp interface between two phases (in this case liquid and solid *γ*. The assumption of local equilibrium implies that the chemical potentials across the interface are the same and that the composition of the elements can be determined from phase diagram information. The rate of the transformation is then solely controlled by the transport of elements towards and away from the interface. Although the assumptions made for these simulations result in a somewhat crude representation of real phase transformations, DICTRA’s strength is its ability to account for multicomponent thermodynamic and diffusion effects. This is achieved by coupled CALPHAD thermodynamic and diffusion mobility descriptions.

**Fig. 4—:**
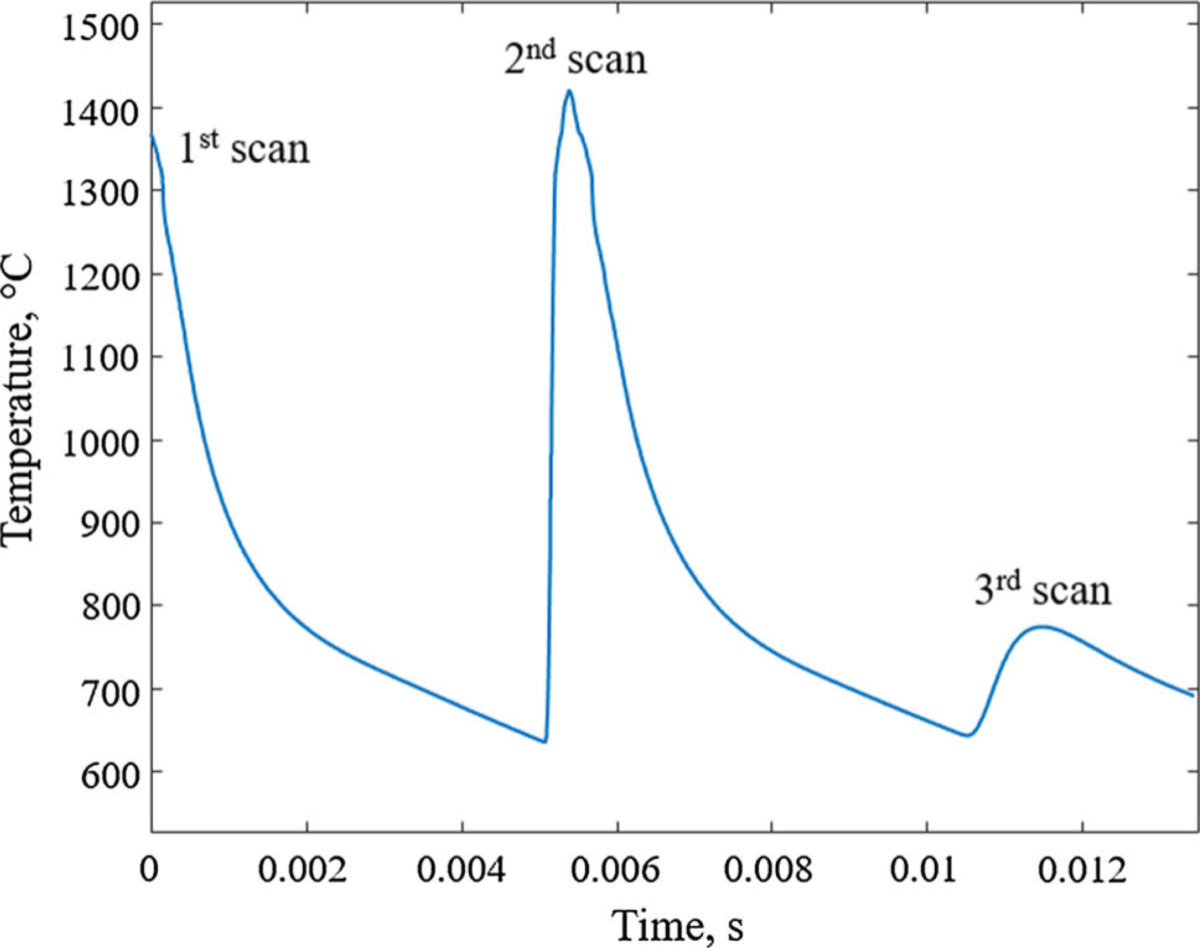
Temperature on the surface of a single layer as a function of time at a position midway between two melt pool centers calculated using a three track scanning FEA thermal model from Ref. [2] This time-temperature profile is used as input for the DICTRA simulation.

In the current work, the commercial CALPHAD database for Ni-based systems, TCNI8/Ni superalloys, is used for the thermodynamic information and the NIST Ni Superalloy mobility database^[22]^ is used for the diffusion coefficient data. The DICTRA simulation domain is 150 nm, which is half the width of the approximate secondary dendrite arm spacing determined from SEM images of the IN625 as-built microstructure.^[2]^ Within the simulation domain, the temperature and pressure of the system are spatially uniform. The global pressure is kept constant at 10^5^ Pa, whereas the temperature evolves with time as calculated using a Finite Element Analysis (FEA) thermal model^[2]^ (Figure 4). The adopted time-temperature profile represents the heat evolution at one point on the surface of a single powder layer during three anti-parallel laser scans 100 *μ*m apart from each other. The point is located midway between the center of two of three anti-parallel laser scan tracks. Details about the FEA thermal modeling can be found elsewhere.^[2]^

### B. Precipitation Simulations

The TC-PRISMA precipitation model^[23]^ is based on the Langer–Schwartz theory^[24]^ and uses the Kampmann–Wagner numerical (KWN) method.^[25]^ It calculates the nucleation, growth, and coarsening of precipitates in multicomponent, multiphase systems. This is enabled through integration of TC-PRISMA with Thermo-Calc and DICTRA and by using CALPHAD descriptions for thermodynamic and diffusion information. The output is the time evolution of the particle size distribution (PSD) and its *n*th moment (*i.e*., number density, mean radius, and volume fraction). Crucial factors for the KWN method are the models for nucleation and growth. Details about the models are found elsewhere.^[23]^ In brief, however, it can be said that the nucleation model in TC-PRISMA is based on the classical theory of nucleation,^[26,27]^ but extended to allow for nucleation in multicomponent systems. Both homogeneous and heterogeneous nucleation can be simulated, where heterogeneities are dislocations, grain boundaries, grain edges, or grain corners. There are two growth models in TC-PRISMA: *advanced* and *simplified*. The *advanced* model accounts thoroughly for high supersaturation and cross-terms in the diffusion coefficient matrix, and can capture transitions between different modes of phase transformation^[28]^ by identifying the operating tie-line from the solution of flux-balance equations. The *simplified* growth model is based on the *advanced* model, but avoids the difficulty of finding the operating tie-line and uses the tie-line across the bulk composition, making the model more efficient to use in terms of calculation time. The *simplified* model is adopted in the current work.

In this work, the TC-PRISMA module in Thermo-Calc version 2017a is used, which is limited to the simulation of spherical particles. Just as in the case of the DICTRA solidification simulations, the thermodynamic information is taken from the TCNI8/Ni superalloy database and the diffusion mobility information is taken from the NIST Ni superalloy mobility database. The precipitates most likely form by way of heterogeneous nucleation, so nucleation on dislocations is assumed in the simulations. Simulations were also performed assuming homogeneous nucleation, and produced similar results, although with different values of the adjustable parameters (*e.g*., interfacial energy). In addition to the nucleation site assumptions, values for the molar volumes and interfacial energies need to be specified when setting up the simulation. Furthermore, the matrix phase, and the phases expected to precipitate need to be pre-defined. In this work, a dislocation density of 5 × 10^11^ m^−2^ is used which corresponds to about 10^21^ m^−3^ nucleation sites. The pre-defined precipitates are δ, $\gamma^{\prime \prime }$, MC, *μ*, and *σ*, and the matrix phase is *γ*. The TCNI8/Ni superalloy database includes descriptions of the molar volumes for the phases and can therefore be calculated directly by TC-PRISMA. The interfacial energies are initially calculated by TC-PRISMA, using the extended Becker model^[29]^ for coherent interfacial energies, but are then scaled so that the values for the different precipitates are, relative to each other, of expected magnitudes. For example, the $\gamma^{\prime \prime }$ -phase is known to have a plate-like morphology. This is due to coherency along the faces and incoherency along the edges. In the case of the *δ*-phase, the interface along the faces is likely semi-coherent. Therefore, the *γ*/*δ* interface is assumed to have a somewhat larger interfacial energy than the $\gamma /\gamma^{\prime \prime }$ interface. The resulting constant interfacial energies that were used in the simulations are 20 mJ/m^2^, 55 mJ/m^2^, 60 mJ/m^2^, 200 mJ/m^2^, and 200 mJ/m^2^ for the $\gamma /\gamma^{\prime \prime }$, *γ*/*δ*, c/MC, *γ*/*μ*, and *γ*/*σ* interfaces, respectively.

## IV. SYNCHROTRON EXPERIMENTS

High-resolution synchrotron XRD experiments were conducted at 11-BM-B at the Advanced Photon Source (APS), Argonne National Laboratory^[30]^ using an X-ray wavelength of 0.414554 Ȧ. XRD data with a 2*θ* step size of 0.00°1 were acquired from 0.5° to 28°. Sample volume was ≈ 1 × 1 × 0.2 mm^3^. The sample was rapidly rotated (3000 RPM) in the beam during data acquisition.

*In situ* synchrotron scattering and diffraction experiments were conducted at the USAXS facility at APS to evaluate the real-time, incipient *δ*-phase growth in the AM-IN625 at 700 °C, 800 °C, and 870 °C. This technique combines *in situ* USAXS, small-angle X-ray scattering, and XRD in the hard X-ray regime,^[31,32]^ and reveals details about precipitation kinetics, including the simultaneous changes in morphology and atomic structure of precipitate phases.^[33]^ The X-ray energy for these measurements was 21 keV, and the X-ray flux was 10^13^ photon/s/mm^2^.^[1,4]^ Volume fractions of precipitate phases are estimated by an XRD peak intensity analysis. For a given peak, the integrated peak intensity is

[1] $$\begin{array}{l} I_{(hkl)\alpha }=\frac{I_{0}\lambda^{3}}{64\pi r}{\left(\frac{e^{2}}{m_{e}c^{2}}\right)}^{2}\frac{M_{\left(\text{hkl}\right)}}{V_{\alpha }^{2}}{\left|F_{(\text{hk}1)\alpha }\right|}^{2}\\ {\left(\frac{1+\cos^{2}(2\theta )\cos^{2}\left(2\theta_{m}\right)}{\sin^{2}\theta \cos\theta }\right)}_{\text{hkl}}\frac{v_{\alpha }}{\mu_{s}}. \end{array}$$

Here, *I*_0_ is the incident beam intensity, *r* is the sample to detector distance, *λ* is the X-ray wavelength, e^2^/m_e_c^2^ is the classical electron radius, *μ*_s_ is the linear attenuation coefficient of the specimen, *M*_hkl_ is the multiplicity of the hkl reflection of phase *α*, *V*_*α*_is the unit cell volume of phase *α*, *v*_*α*_ is the volume fraction of phase *α*, 2*θ*_m_ is the diffraction angle of the monochromator, and ${\left(\frac{1+\cos^{2}(2\theta )\cos^{2}\left(2\theta_{m}\right)}{\sin^{2}(\theta )\cos(\theta )}\right)}_{hkl}$ accounts for the Lorentz and polarization corrections.

Following Eq. [1], the intensity ratio between a precipitate peak and a matrix peak is, therefore,

[2] $$\frac{I_{(hkl)p}}{I_{(hkl)m}}=\frac{M_{(hkl)p}V_{m}^{2}}{M_{(hkl)m}V_{p}^{2}}{\left|F_{(hkl)m}\right|}^{2}\frac{{\left(\frac{1+\cos^{2}(2\theta )\cos^{2}\left(2\theta_{m}\right)}{\sin^{2}\theta \cos\theta }\right)}_{(hkhp}\frac{v_{p}}{v_{m}}}{{\left(\frac{1+\cos^{2}(2\theta )\cos^{2}\left(2\theta_{m}\right)}{\sin^{2}\theta \cos\theta }\right))}_{(hkl)m}}.$$

Here, *m* and *p* indicate matrix and precipitate, respectively. Equation [2], thus, allows the volume fraction of *v*_p_ to be estimated.

## V. RESULTS AND DISCUSSION

### A. Microsegregation During Solidification

**Fig. 5—:**
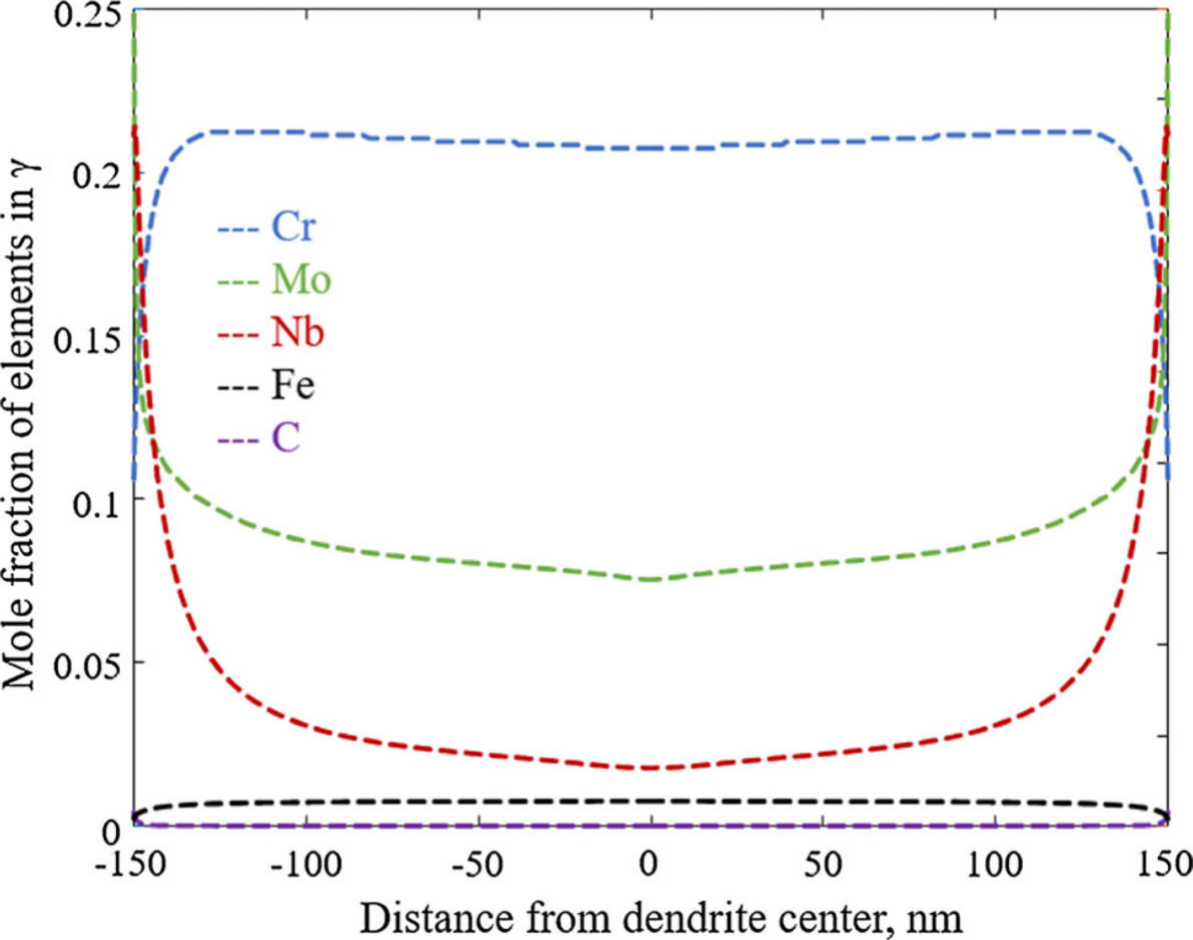
Composition profiles in *γ* as predicted by the DICTRA simulation using the thermal history presented in Fig. 3 as a function of distance from the secondary dendrite core (*x* = 0).

The DICTRA solidification simulation starts at a temperature above the liquidus of the system. This is the first peak temperature shown in Figure 4 and corresponds to the laser passing the tracked point the first time. At this time step, the whole DICTRA simulation domain is thus a homogenous liquid. When the laser passes the point during a second anti-parallel scan, the domain completely re-melts as the temperature reaches about 1700 K (second peak in Figure 4). During the third scan (third peak in Figure 4), further away from the point, the temperature is only increased to about 1050 K and the domain, which is fully solidified after the cooling down from the second scan, does not re-melt again. The distribution of alloying elements over the secondary arm spacing produced from these heating and cooling cycles is shown in Figure 5. Closest to the last solidified liquid (at *x* = − 50 nm and *x* = 150 nm in Figure 5), the microsegregation is the largest. The interdendritic regions are, in particular, observed to be enriched in Mo and Nb and depleted in Cr. Carbon also segregates towards the solidification front, whereas Fe segregates away from the solidification front. These solidification characteristics are in accordance with the experimental observations where energy dispersive spectroscopy (EDS) maps have shown enrichment of Nb and Mo as well as Cr depletion in interdendritic regions.[1,2]

The DICTRA simulation is an approximate representation of the AM process and several simplifications and assumptions are made such as assuming 1D planar solidification and local equilibrium at the interface. The latter assumption, in particular, is limiting when studying solidification at high cooling rates since deviations from local equilibrium due to finite interface kinetics and solute trapping are expected. By enforcing local equilibrium without accounting for trapping effects, the DICTRA simulation therefore overestimates the extent of segregation. In this aspect, phase-field solidification models that allow for both curved interfaces and solute trapping could be a way to go to produce estimations of microsegregation during AM, see, *e.g*., References 34–37. Uncertainties are also introduced by the applied time-temperature profile which is not as complex as could be expected for AM and by the estimation of the secondary arm spacing. However, in the following discussion, the calculated microsegregation shown in Figure 5 is assumed to be representative for an arbitrary location in the as-built IN625 microstructure.

**Fig. 6—:**
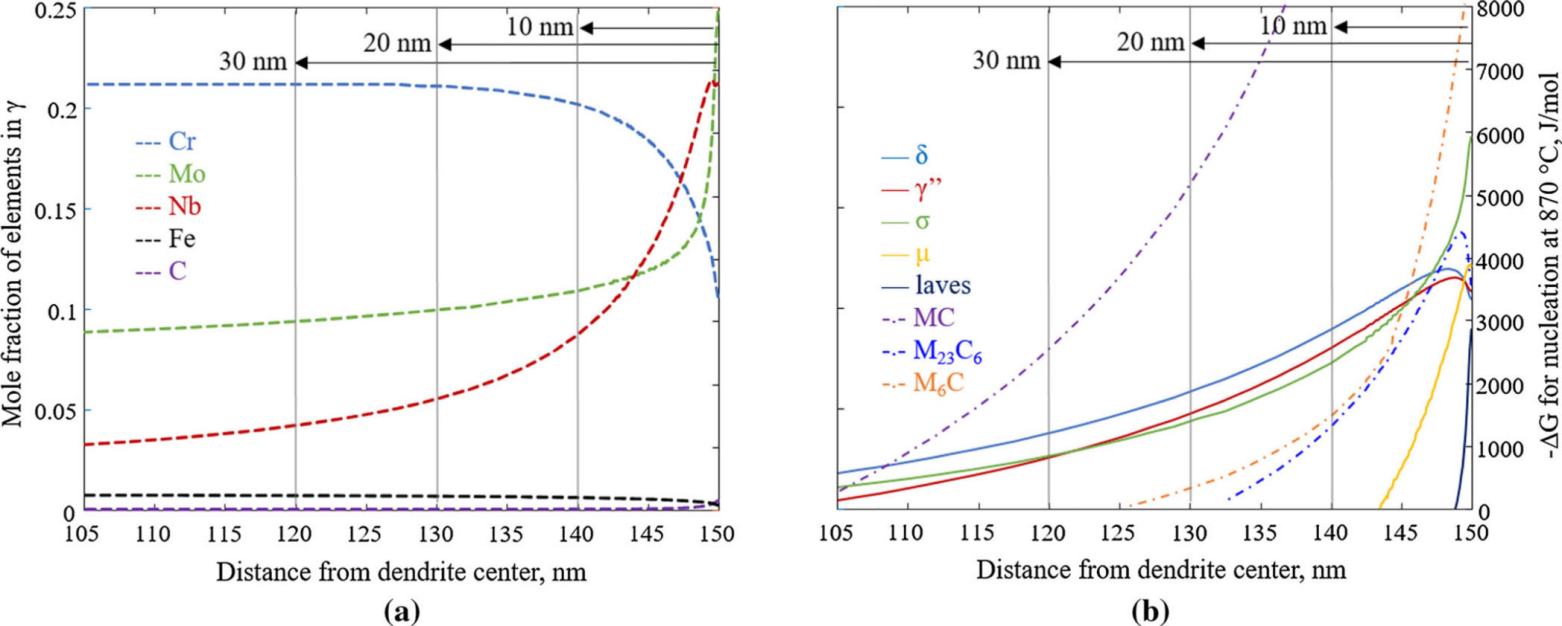
(*a*) Same data as in Fig. 5 showing the region closest to the interdendritic region center. The compositions selected for the TC-PRISMA simulations are marked at 30 nm, 20 nm, and 10 nm from the interdendritic region center. (*b*) Corresponding calculated nucleation driving forces, –Δ*G*, for the phases *δ*, *σ*, *μ*, laves, MC, M6C, and M_23_C_6_ at 870 °C using the TCNI8 thermodynamic database.

Figure 6(a) shows the composition profiles as a function of distance in the vicinity of the interdendritic region. To investigate how the segregated as-built microstructure at these compositions may react upon heat treatment, the driving force, Δ*G*, for precipitates to nucleate from a *γ* matrix is calculated at 870 °C. Note that contributions from interfacial effects and kinetic obstacles are ignored. The results are shown in Figure 6(b) and include the phases of interest that showed the largest driving forces, *i.e*., MC, M_23_C_6_, M_6_C, *σ*, *μ*, laves, *δ*, and $\gamma^{\prime \prime }$. In the figures, –Δ*G* is plotted, and thus values greater than 0 indicate that nucleation from the *γ* matrix is thermodynamically favorable, whereas values less than 0 indicate non-favorable nucleation. In Figure 6(b), only values greater than 0 are shown. As the enrichment of Nb and Mo increases, when moving from the dendrite arm core towards the interdendritic region, the driving forces for all the phases increase. Hence, from a thermodynamic point of view, precipitation of all of the phases considered become more favorable in the segregated regions due to the increase in Mo and Nb composition. The driving forces for *δ*, $\gamma^{\prime \prime }$, and *σ* are particularly of comparable magnitude and, depending on process conditions, competitive precipitation of these phases is expected. The driving force for the laves phase becomes comparable to the *δ* and $\gamma^{\prime \prime }$ driving forces at the very center of the dendrite; *i.e*., according to the calculations, it is not thermodynamically favorable for laves to form unless the segregation is sufficiently severe.

### B. Precipitation During Post-treatments

Although the nucleation driving force is a useful quantity, when trying to elucidate how a microstructure may evolve, the kinetic aspects and interfacial energy contributions may play a decisive role. From the calculated equilibrium phase diagram in Figure 3(a), as well as from experimental observations, it is known that the *δ*-phase is an equilibrium phase at the industry-recommended stress-relieving temperature 870 °C for the nominal IN625 composition. However, the nucleation and its growth are too slow for it to reach a detectable volume fraction within the suggested heat treatment time (1 hour) in the case of wrought IN625 as shown by the experiments by Floreen *et al*. ^[9]^ and Suave *et al*.^[10]^ To study the kinetics of the precipitation during heat treatment of AM IN625, and how it may alter locally throughout the as-built, highly segregated microstructure, TC-PRISMA simulations are performed for compositions at different locations along the dendrite arm spacing, *i.e*., at 30 nm, 20 nm, and 10 nm from the interdendrite region center, see Figure 6(a). The phases *δ*, $\gamma^{\prime \prime }$, *σ*, *μ*, and MC are included in the simulations. Although the laves phase has the same constituents as *δ* and thus could be an expected phase, it is not included in the simulations. The reason for this is that its driving force for nucleation is much smaller than the included phases at the selected composition as shown in Figure 6(b); *i.e*., even if laves would be included in the simulation, it would not form. Further, the laves phase is also not experimentally observed in the current work. For the simulations, the C content is kept the same in all simulations, i.e., at a mass fraction of 0.02 pct. Only isothermal precipitation kinetics are considered; *i.e*., heating and cooling are not included. Simulations are performed at several temperatures to enable the construction of TTT diagrams.

**Fig. 7—:**
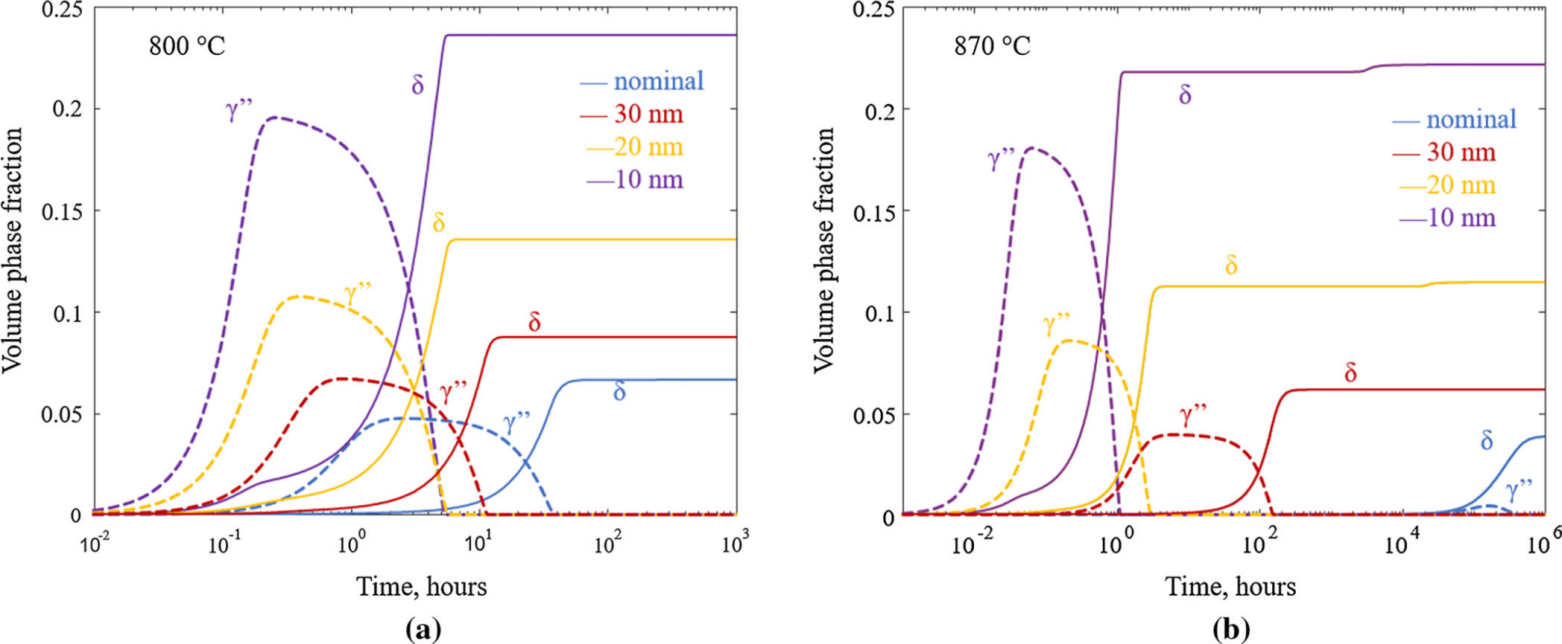
Calculated volume phase fractions at (a) 800 °C and (b) 870 °C for the nominal composition (blue lines), and the compositions at 30 nm (red lines), 20 nm (yellow lines), and 10 nm (purple lines) from the interdendritic region center (Color figure online).

The results of the TC-PRISMA simulations at 800 °Cand 870 °C in terms of volume fraction as a function of time are shown in Figures 7(a) and (b), respectively. For both temperatures and all compositions, the MC carbides precipitate first due to its high driving force for nucleation. The volume fraction of the phase is determined by the amount of C, and hence, it is the same for all simulations and reaches its maximal fraction value almost immediately (not visible in Figure 7(a) and (b). After precipitation of MC, $\gamma^{\prime \prime }$ precipitates followed by *δ* precipitation for all simulations. Evidence that the *δ*-precipitation in AM IN625 may be preceded by $\gamma^{\prime \prime }$ precipitation is reported in Reference 3 and the competition between $\gamma^{\prime \prime }$ and *δ* in the interdendritic regions is further discussed by Lass et al.^[17]^ For the nominal composition (blue lines, Figure 7), the formation of the $\gamma^{\prime \prime }$ -phase is more pronounced at lower temperature (Figure 7(a): dashed blue line) compared to higher temperature (Figure 7(b): dashed blue line) which is in accordance with experimental observations for wrought IN625.^[9]^ The volume fraction of the precipitates also increases with decreasing temperature which is expected from the equilibrium calculations (Figure 3(a)).

When *δ* starts to form, the fraction of $\gamma^{\prime \prime }$ begins to decrease, and before the equilibrium fraction of d is reached, the $\gamma^{\prime \prime }$ precipitates have completely dissolved. This can be explained by $\gamma^{\prime \prime }$ being metastable (in the IN625 system). As discussed in the previous retion, the driving force for the $\gamma^{\prime \prime }$ and *δ* precipitates to nucleate from the *γ* matrix increases with increasing degree of segregation (mainly due to enrichment of Nb and Mo) which, consequently, contributes to earlier precipitation kinetics in segregated areas. For example, for the nominal compositionat800 °C, the *δ*-phase has reached a volume fraction of 1 pct after 13.5 hours, whereas for the composition 10 nm from the center of the inter dendrite region, avolume fraction of 1 pct is reached after 8 minutes.

**Fig. 8—:**
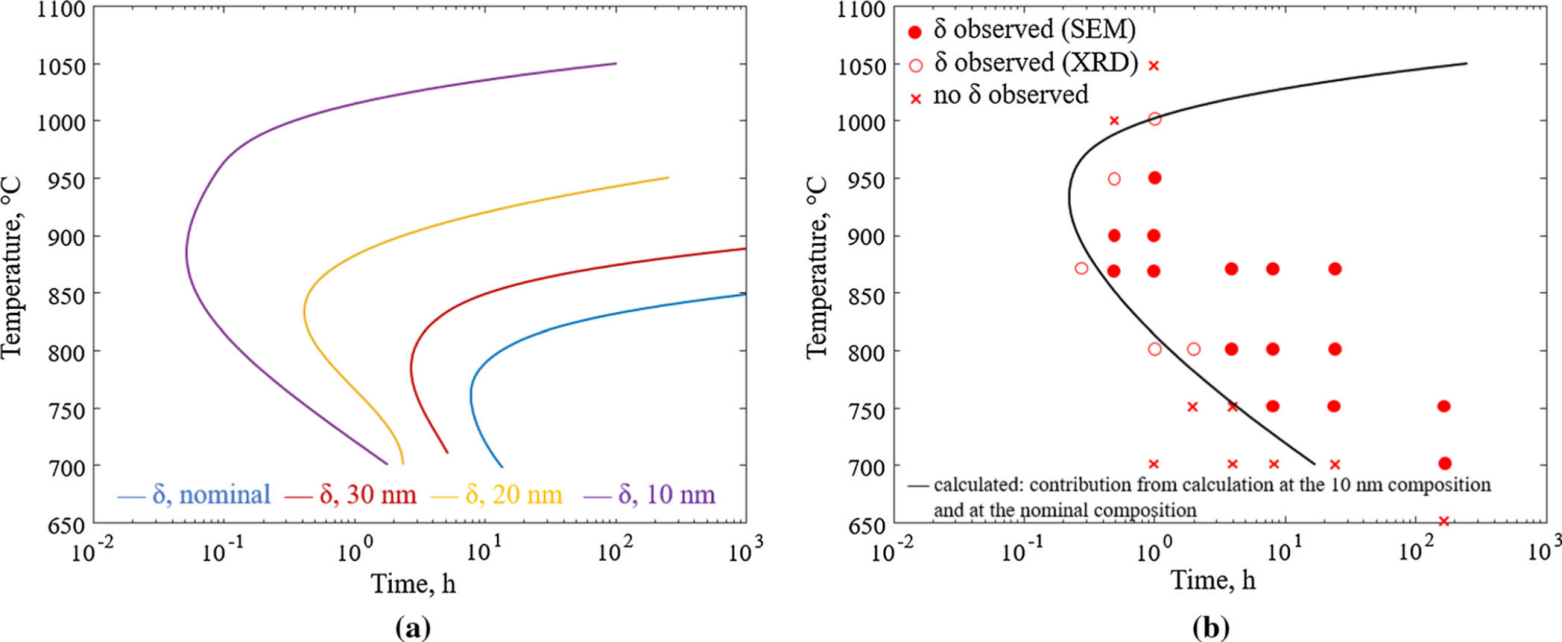
(*a*) Calculated TTT curves showing the time-temperature combinations that result in a volume fraction of 1 pct *δ*-phase for the nominal composition (blue line), and the compositions at 30 nm (red line), 20 nm (yellow line), and 10 nm (purple line) from the interdendritic region center. (*b*) Calculated TTT curve showing the time-temperature combinations that result in a volume fraction of 1 pct *δ*-phase assuming contributions from the interdendritic region and regions of compositions similar to the nominal composition (solid line) in comparison with the experimentally determined TTT diagram from Refs. [3, 5] (Color figure online).

TC-PRISMA simulations are performed at several temperatures for the different segregated compositions and are presented in Figure 8(a) in terms of a TTT diagram. For each composition, the curves indicate the temperature-time combinations for which the d-phase has grown to a volume fraction of 1 pct. From the TTT diagram, it can be concluded that the precipitation of the d-phase, in addition to occurring at a shorter timescale, also is extended to higher temperatures with increasing segregation. While it takes thousands of hours for the *δ*-phase to form in the case of the nominal composition (blue solid curve in Figure 8(a)) at 870 °C, the *δ*-phase forms during early stages (seconds to minutes) up to over 1000 °C in the case of the 10 nm composition (purple curve in Figure 8(a)). Exact agreement with the experimental TTT curve is not expected due to the model assumptions and the use of the reduced composition. Yet the present calculations support the hypothesis that the accelerated precipitation kinetics observed in AM IN625 is due to local, extreme, compositional changes due to the microsegregation.

Since each calculated curve in the TTT diagram (Figure 8(a)) only represents one point in the microstructure, a single curve alone cannot describe the precipitation behavior in AM IN625. Instead, integrating over all the compositions would result in a volume phase fraction evolution more justifiably comparable to experimental observations. Performing TC-PRISMA calculations at all compositions in the segregation profile is, however, beyond the scope of this work and simulations are only performed at select compositions. Nevertheless, a simplified comparison is made by weighing the contribution of the different simulations to the overall volume fraction evolution differently depending on location. Here, it is assumed that the interdendritic regions represent about 20 pct of the microstructure and can be represented by the simulations for the composition at 10 nm from the interdendritic center, Figure 6(a). The remaining part of the microstructure is assumed to be represented by the simulation for the nominal composition. The resulting TTT curve is shown in Figure 8(b) and is comparable with the experimentally determined TTT diagram.

**Fig. 9—:**
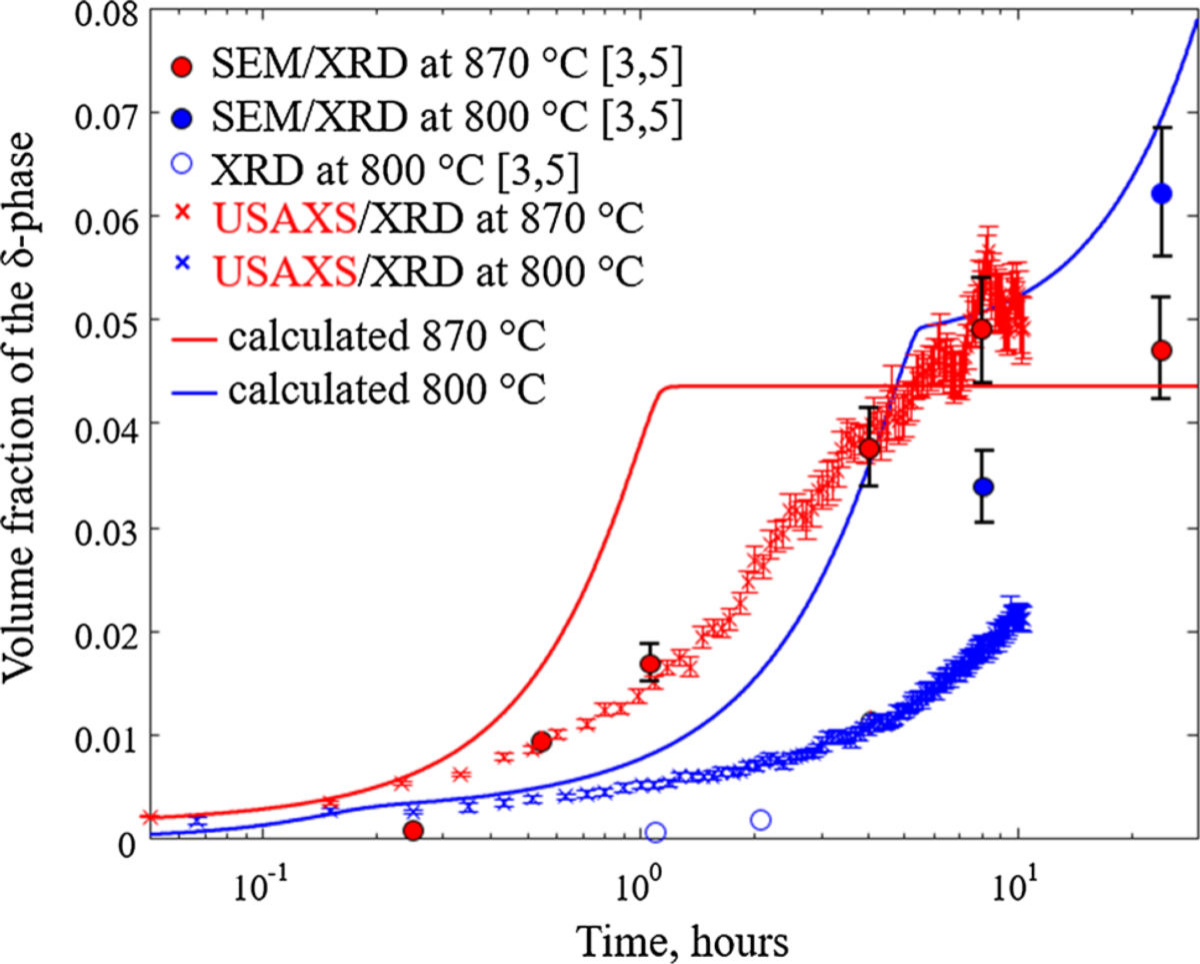
Calculated volume fraction of the d-phase as a function of time assuming contributions from the interdendritic region and regions of composition similar to the nominal composition at 800 °C (blue solid line) and 870 °C (red solid line), in comparison to experimentally measured volume fractions by SEM/XRD (from Refs. [3, 5]) and USAXS/XRD. Open blue circles correspond to measurements with large uncertainty due to the difficulties in estimating low-volume fractions from SEM images. The error bars associated with each data point represent an estimated 15 pct uncertainty (Color figure online).

In Figure 9, the evolution of the volume fraction of the *δ*-phase with time is shown for the simulations at 800 °C and 870 °C, in the case of the assumption described previously (contribution from the nominal and the 10 nm composition). The volume phase fractions determined experimentally, both by SEM and laboratory XRD,^[5]^ and by USAXS/XRD, are also included. Some discrepancy in the two sets of experimental data is seen. Measuring phase fractions from SEM images leads to uncertainties, particularly at low *δ* fractions since it is difficult to separate between the *δ*-precipitates and the Mo-Nb-rich interdendritic regions using backscatter electron imaging or EDS. In addition, the *δ*-precipitates are thin, only about 30 nm to 50 nm thick, and resolving that thin of a precipitate to a level that provides highly accurate phase fractions is difficult. The USAX/XRD measurements are more quantitative since they directly measure phase fractions throughout the measured sample volume of approximately 1.2 × 0.8 × 0.03 mm^3^, and these measurements probed the same sample volume *in situ* during the isothermal anneals.

Nevertheless, comparison of the simulations with the experimental observations shows qualitative agreement. At 870 °C, both the simulations and experiments predict that the volume fraction is approaching a constant with increasing time. At 800 °C, both the simulations and experiments show the volume fraction increasing as function of time. The discrepancy between the experiments and simulations is in how fast the volume fraction of *δ* increases: the simulation predictions are much faster than what is observed. At 870 °C, the equilibrium volume fraction of *δ* is reached at approximately 1 hour; however, experimentally the volume fraction does not begin to plateau until 7 hours.

The simulations only assume contributions to the precipitation kinetics from compositions at two locations and do not average over all the compositions in the microstructure. In addition, the precipitation simulation using TC-PRISMA is based on a number of assumptions and approximations including uncertainties in the classical theory of nucleation, the choice of nucleation sites, interfacial energies, and in the thermodynamic and diffusion mobility databases. Small changes in input data and nucleation assumptions could alter the simulation outcome. Despite these shortcomings, the ability of the calculations to reproduce the experimental TTT curve, and qualitatively assess certain characteristics of the precipitation during post-processing, motivates exploring the possibility of using simulations to guide the selection of a stress-relieving treatment.

**Fig. 10—:**
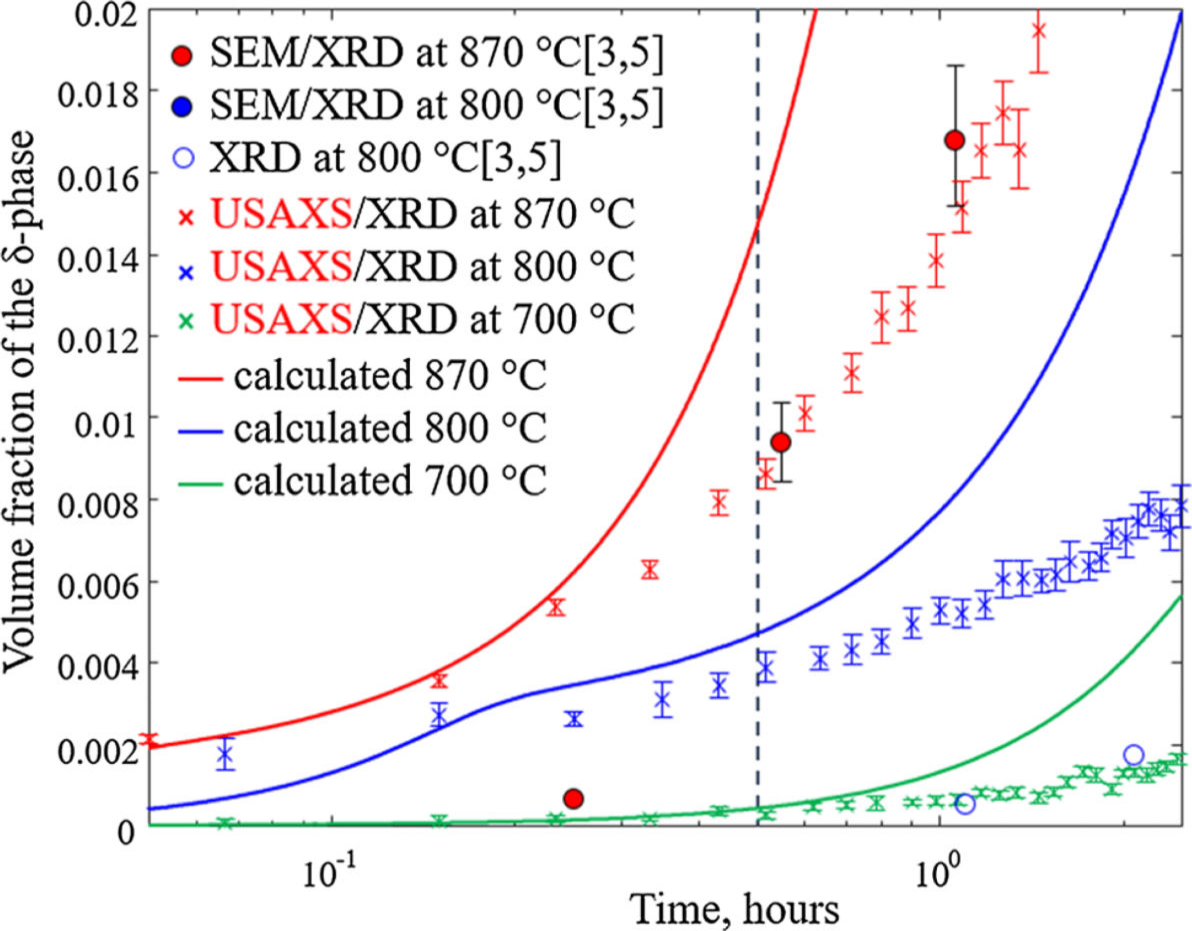
Calculated volume fraction of the *δ*-phase as a function of time assuming contributions from the interdendritic region and regions of composition similar to the nominal composition at 700 °C (green line), 800 °C (blue line), and 870 °C (red line), compared to experimentally measured volume fractions by SEM/XRD (from Refs. [3,5]) and USAXS/XRD. Open blue circles correspond to measurements with large uncertainty due to the difficulties to estimate low-volume fractions from SEM images. The error bars associated with each data point represent an estimated 15 pct uncertainty. The dashed line indicates the *x*-value 0.5 h (Color figure online).

In Figure 10, the calculated phase fraction evolution is shown for the initial heat treatment (up to 2.5 hours) at 700 °C, 800 °C, and 870 °C. In comparison, the experimental results by Stoudt et al.^[5]^ (800 °C and 870 °C) are shown as well as the USAXS/XRD results (700 °C, 800 °C, and 870 °C). According to the experimental work by Lass and Stoudt *et al*.,^[3,5]^ the industry-recommended stress-relieving at 870 °C for an hour results in a volume fraction of about 2 pct *δ*-phase. If the stress-relieving temperature instead is lowered to 800 °C, they found that a volume fraction of less than 0.5 pct *δ*-phase forms during the first hour of heat treatment. Since IN625 reaches its optimal strength when as much alloying elements as possible are dissolved in the c solid solution, a stress-relieving treatment that minimizes the *δ*-phase formation while removing residual stresses, and hence enables for efficient homogenization, should be aimed for. Thus, Lass *et al*.^[3]^ recommended the stress-relieving temperature of 800 °C. The present simulations support this suggestion. Despite the discrepancy with regard to the measured phase fraction, the calculated phase fraction as a function of time in Figure 8 indicates that a stress-relieving at 870 °C for an hour would result in the formation of about four to five times as much *δ* compared to stress-relieving at 800 °C. For long-term treatment, however, heat treating at 800 °C will result in a larger *δ*-phase fraction. This is expected from the phase diagram for the IN625 nominal composition where the equilibrium phase fraction of d increases with decreasing temperature (Figure 4). This is also in accordance with experimental findings for L-PBF IN625 (Figure 8).^[5]^ Heat treating at 700 °C would result in even less *δ* formation. However, relieving the stresses at such a low temperature requires longer holding times, and hence, more time for *δ* to grow together with increased processing time and cost.

## VI. CONCLUSIONS

This work evaluates the ability to use common computational thermodynamic and kinetic tools (Thermo-Calc, DICTRA, and TC-PRISMA) to study the microstructure evolution in IN625 manufactured using the AM technique L-PBF. Experimental observations and DICTRA solidification simulations indicate that laser melting and re-melting during L-PBF produce highly segregated interdendrite regions. This, in turn, results in accelerated *δ* precipitation during subsequent heat treatments compared to wrought IN625. We established that an increase in the Nb and Mo concentration leads to the increase of nucleation driving forces, making precipitation of *δ*, $\gamma^{\prime \prime }$, and MC carbide phases more favorable in the segregated regions. Precipitation simulations using TC-PRISMA for different degrees of segregation and at different temperatures enabled construction of a TTT diagram for AM IN625. It is concluded that the larger the segregation, *i.e*., the richer the interdendritic regions are in Nb and Mo, the faster the *δ* precipitation. Furthermore, the stability of the *δ*-phase shifts to higher temperatures with increasing segregation. Assuming that the overall phase evolution consists of contributions from both segregated areas and areas with compositions closer to the nominal IN625 composition, the calculated results are comparable to the experimentally determined TTT diagram. In accordance with experiments, the calculations suggest that stress-relieving at 800 °C would limit the *δ* formation to about 20 pct to 25 pct of the volume fraction formed during stress-relieving at 870 °C, and hence would facilitate more efficient homogenization. The simulations also suggest that *δ* may form at temperatures as high as 1050 °C which, consequently, should be considered when selecting a homogenization heat treatment.

## Figures and Tables

**Table I.** The Measured, Nominal Composition in Mass Fraction Pct of the Powder Used for the AM of the IN625 Part Studied Experimentally (Refs.^3^ through 5) and the Reduced Composition Used for the Calculations

Element	Composition According to Delivery Certificate	Reduced Composition Used for the Calculations
Ni	balance	balance
Cr	20.7	20.7
Mo	8.83	8.83
Nb	3.75	3.75
Fe	0.72	0.72
Ti	0.35	—
Al	0.28	—
Co	0.18	—
Si	0.13	—
Mn	0.03	—
C	0.01	0.02
P	0.01	—
S	0.002	—

## Footnotes

* Certain commercial entities, equipment, or materials may be identified in this document in order to describe an experimental procedure or concept adequately. Such identification is not intended to imply recommendation or endorsement by the National Institute of Standards and Technology (NIST), nor is it intended to imply that the entities, equipment, or materials are necessarily the best available for the purpose.
